# “Give Me Proof”: A Covert but Coercive Form of Non-partner Sexual Violence Contributing to Teen Pregnancy in Haiti and Opportunities for Biopsychosocial Intervention

**DOI:** 10.1080/10926771.2020.1738616

**Published:** 2020-04-09

**Authors:** Guitele J. Rahill, Manisha Joshi, Caron Zlotnick, Sabine Lamour, Haley Beech, Amber Sutton, Cameron Burris, Phycien Paul

**Affiliations:** a School of Social Work, CBCS, University of South Florida, Tampa, FL, USA; b Department of Psychiatry and Human Behavior, Medicine, and Ob/Gyn, Brown University, Providence, RI, USA; c SOFA, Solidarite Fanm Ayisyen/Solidarity of Haitian Women, Port-au-Prince, Haiti; d Social Work, University of Alabama School, Tuscaloosa, AL, USA; e Centre for Women, Tampa, FL, USA; f OREZON Cité Soleil, Port-au-Prince, Haiti

**Keywords:** Adolescent pregnancy in Haiti, gendered vulnerability, coercive control, human rights and reproductive health, trauma, depression and suicide, 2010 Haiti earthquake

## Abstract

Adolescent girls in low-resource settings account for over 7.3 million births annually (generally unplanned). Unplanned teen pregnancies are increasing in low-resource settings. As part of a funded Round 20 Grand Challenges Exploration project (Healthy Minds for Adolescent Mothers), we investigated unplanned teen pregnancies in Haiti’s Cité Soleil shantytown, teens’ biopsychosocial challenges, and desirable interventions. Key stakeholders (N = 23): pregnant teens (13-17-year-olds, n = 8; 18-19-year-olds, n = 8) and health providers (18 or older, n = 7), participated by age group and role in focus groups (FGs). ATLAS.ti facilitated the analysis of transcribed FG audio recordings. Ninety-four percent (n = 15) of teens reported “Banm prèv,” translated “Give me proof,” as a cause of their unplanned pregnancies. Banm prèv describes when teens are propositioned by men who demand proof of their unpretentiousness or virginity. A subtle, covert, locally unchallenged phenomenon that is supported by damaging gender norms, Banm prèv constitutes an illusionary choice between teens’ yielding non-consensual control of their bodies and the tacit alternative of gang rape. Banm prèv underscores teens’ difficulty discerning consensual from coerced sex. Associated unplanned pregnancies occasion guilt, shame, stigma, depression, anxiety, and trauma in teens. Cité Soleil teens need contextually relevant, community-supported, age-appropriate interventions that challenge existing norms, build on cultural strengths, and include comprehensive sexuality education, including knowledge of reproductive rights. A traditional, contextually familiar, engaging, and humorous story-telling tradition, i.e., krik-krak, packaged in video format, is a useful framework for interventions to reduce depressive symptoms, stress, and anxiety for Cité Soleil teens experiencing unplanned pregnancies.

## Introduction

The Sustainable Development Goals of 2030 include empowering all persons to live healthy lives and ensuring the health and welfare of all persons across the lifespan (United Nations Development Programme, 2018). Girls and women who are safe from violence are critical to building a healthier world and to raising educated and successful children who can take their rightful place as global citizens. But, in the most resource-limited enclaves of low- and middle-income countries (LMICs), the health of girls and women is often endangered by poverty and gender-based violence (GBV), especially during adolescence, when teens in such settings face unplanned pregnancies (United Nations Population Fund [UNFPA], 2018; World Health Organization [WHO], 2018a).

Teens in LMICs account for over 7.3 million births annually, resulting mostly from unplanned and unwanted pregnancies; these figures are expected to increase (UNFPA, 2018). Over 800 girls and women die in pregnancy or childbirth daily, typically due to lack of interventions (WHO, 2018a).

Unplanned teen pregnancies represent barriers to teens’ ability to continue their education and, thus, an obstacle to their future economic independence (UNFPA, 2018). Unplanned teen pregnancies also occur during a period of mood fluctuations that include depression and anxiety which compound hormonal and emotional changes in teens (WHO, 2018a, 2019). Depression during pregnancy is a risk factor for girls and women becoming victim to intimate partner violence (IPV) and to non-partner sexual violence (NPSV). When depression occurs during an unplanned teen pregnancy, it further hinders the expectant teen’s ability to protect and to care for herself (WHO, 2019). Depression during an unplanned teen pregnancy can also be detrimental to the teen’s post-partum health, jeopardize her ability to care for her infant, and negatively impact her child’s cognitive development (Global Grand Challenges [GCC], 2017). In some cases, maternal depression, especially in teens who may lack emotional regulation capacities, can result in suicide and infanticide (Vigod & Stewart, 2017).

Recognizing teen pregnancy as a human rights issue, UNFPA enhances access to sexual and reproductive health in LMIC contexts, and collaborates with local gatekeepers to improve adolescents’ access to education, comprehensive sexuality education, and health care (UNFPA, 2018). But, in resource-limited Haiti, prevention of teen pregnancy and the health of pregnant teens are often neglected due to lack of resources. The 2010 earthquake and 2016’s Hurricane Matthew greatly reduced the nation’s financial capacity to manage the constant threat of preventable infectious diseases and to help those who are still recovering from the devastating impact of the 2010 earthquake and from the trauma associated with the ensuing ravages of Hurricane Matthew (Interagency Working Group on Reproductive Health in Crises, 2016). Moreover, over ¾ of individuals in LMICs such as Haiti who need mental health care do not receive it (WHO, 2019). Thus, teen pregnancy is not among the nation’s top priorities. Yet, teen pregnancy in Haiti should be a priority for several reasons. First, Haiti is one of eight nations that have the highest risk of teens dying from pregnancy or childbirth (Gunawardena et al., 2019). Second, little is known about the health and mental health of teens in Haiti because they are underrepresented in research (Eustache et al., 2017). Third, Haitian females have a fourfold higher rate of Major Depressive Disorder compared to Haitian men (Eustache et al., 2017). Fourth, roughly 25% of pregnant women and nearly 20% of new mothers globally experience depression, and teens with depression have a higher suicide risk (WHO, 2019). Fifth, suicide is the principal cause of death for teens younger than 19 years of age, and over 90% of teen suicides globally occur in LMICs such as Haiti (GCC, 2017; UNFPA, 2018; WHO, 2019).

### Purpose of the study

We investigated factors leading to unplanned teen pregnancies in urban Cité Soleil, a shantytown in Haiti’s Port-au-Prince (Hereafter, CS) and the biopsychosocial challenges and outcomes of such pregnancies. Our work was facilitated by a grant procured during the Grand Challenges Exploration Round 20 competition, funded by the Melinda Gates Foundation (OPP1191065). To our knowledge, this is among the first studies conducted in Haiti that obtains the perspectives of pregnant teens and health providers (Hereafter HPs) concerning unplanned teen pregnancies in Haiti and associated biopsychosocial challenges and outcomes. In accomplishing the objectives of this study, we add to the extant literature regarding the health and mental health of pregnant teens in resource-limited settings.

## Study setting: Cité Soleil (CS)

Cité Soleil, literally “Sun City,” is a seaside shantytown in Port-au-Prince Haiti. Most Cité Soleil residents are peace-loving, respectful, and friendly. CS adults strive to improve their children’s quality of life through educational opportunities. Indeed, CS schoolchildren received a 93% passing rate on Haiti’s national exam for the 2018–19 school year, exceeding the performance of their peers in more socially advantaged neighborhoods; a library under construction promises to maintain this excellence (*Ministère de l’Education Nationale et de la Formation Professionnelle* [National Ministry of Education and Professional Training], 2019). CS is replete with bright, educated, and capable, albeit young leaders. Several non-governmental organizations (NGOs) endeavor to provide education and reduce various forms of violence (Torgan, 2019). However, lack of collaboration among NGOs, political exclusion of CS residents, and diversion of funds directed for CS to corrupt leaders, reduce the impact of well-intended efforts in the area (Kolbe, 2015).

CS is densely populated, with over a quarter of a million residents (nearly 400,000, roughly 50% females) living in just over 20 square kilometers (Konbit Soley Leve, n.d.). The average salary of residents is less than 1 USD.25 per day, lower than the national average of 2 USD per day (World Bank, 2019). Consistent with the average age of 25 in Haiti, CS has a young population, the averaging 15 to 35 years old (Sakala, 2019). Due to a confluence of poverty, hunger, malnutrition, widespread violence, and preventable infectious diseases, CS residents rarely attain age 50, 2 years below the national life expectancy of 52 (Konbit Soley Leve, n.d.). Moreover, CS is socially and politically isolated and poorly policed, permitting various types of violence, including GBV, to perpetuate with impunity (United States Agency for International Development [USAID], 2017b). Even the United Nations forces often avoid the CS due to violence (Faedi, 2008).

### GBV in Cité Soleil

GBV is not unique to Haiti and increases in times of political strife, during regional conflict, and in the aftermath of disasters. Indeed, a third of girls and women around the globe have experienced some sort of physical or sexual violence (UNFPA, 2018). GBV toward girls and women in CS sometimes comes from outside the neighborhood, stemming from societal stigma regarding poor people and devaluation of the neighborhood’s residents (Kolbe, 2015). GBV rates in CS more than doubly exceed global figures, often increasing when members of various gangs randomly rape girls and women as part of turf wars (Faedi, 2008). Depending on the source, between 50% and 72% of CS girls and women experience intentionally brutal, coercive, and misogynistic acts of NPSV committed by several aggressors; these rapes are reportedly aimed at first inundating, then destroying their uterus (Faedi, 2008; Joshi et al., 2014). Yet, violence experienced by girls and women in CS and adjacent shantytowns is not prioritized for several reasons, including gender-based inequities that minimize their biopsychosocial needs and exclude them from politics and positions of power (Faedi, 2008; USAID, 2017a). Besides, perpetrators of NPSV in CS are rarely punished since the burden of proof falls on the NPSV victims and their parents (Faedi, 2008).

The occurrence of GBV in CS is relevant because adolescent risk for mental health problems is directly proportional to the number of adverse personal, social, and environmental conditions (WHO, 2019). Ostensibly, enduring recurrent gang violence and experiencing, witnessing, or hearing about GBV and NPSV in CS can heighten resident teens’ vulnerability to stress and trauma-related disorders such as post-traumatic stress disorder (PTSD). Relatedly, PTSD during adolescence impedes teens’ ability to develop a strong and resilient identity, limiting their ability to cope with new stressors. Moreover, suicide risk is higher in persons with PTSD, who may have intrusive memories, anger, and poor impulse control (American Psychiatric Association, 2013). Accordingly, for a Haitian teen in CS who experiences an unplanned pregnancy, the likelihood of depression, anxiety, PTSD, and suicide may be greater compared to non-pregnant peers who experience similar environmental and contextual conditions (Eustache et al., 2017).

## Theoretical framework

Coercive Control, Gendered Vulnerability, and Human Rights are useful lenses for investigating unplanned teen pregnancies and associated challenges in CS . Coercive control refers to the assertion of power over female victims of violence through “isolation, intimidation, threats, withholding of necessary resources …, [which may be exacerbated by] psychological, physical, or economic vulnerability of the target of control” (Dutton et al., 2006, p. 1, 50). Relatedly, gendered vulnerability refers to the “emotional, cognitive, behavioural, social, or physiological conditions that reduce [a woman’s] capacity or likelihood of resistance to coercion” (Dutton et al., 2006, p. 1). Gendered vulnerability is a global issue that especially affects poor women; it is “a main reason why over a billion children suffer extreme poverty, because [vulnerable] women everywhere are assigned primary responsibility for caring for children, a responsibility that helps to impoverish both groups” (Jaggar, 2009, p. 33).

Lamour addresses the control and gendered vulnerability in the Haitian context. She reports that gender-based roles in Haiti are intentionally constructed so that from early childhood, boys are socialized to be concerned with their own needs and to enjoy their personal and financial resources while girls are socialized to accept that their bodies are a resource to be possessed, much as land (Lamour, 2019). She maintains that throughout the socialization process, girls are taught to accept male and societal domination, while being given a strong sense of responsibility for and accountability to others, and, being consigned as the *poto mitan* of the family (literally the center post, figuratively “load bearing post of a structure” (Bergan & Schuller, 2009).

Human rights suggest that Haitian teens, as described in the present study, should have a clear understanding of consent and of their right to refuse sex or particular sexual partners. It also means that teens should have the autonomy regarding when they engage in sexual interaction, when they become pregnant and by whom they become pregnant. Human Rights in the present study further means that teens should expect and be guaranteed personal and legal protection from NPSV of any form, have the same access to contraception as boys and men, and have access to culturally and age-appropriate interventions that address depressive, stress, anxiety, and trauma-related disorders (The Lancet Commissions, 2018).

## Methods

We used a qualitative design and purposive sampling methods to recruit 23 participants who would participate in focus groups (Hereafter, FGs) based on their ages or roles (teens; HPs). A sample size of 23 is sufficient in qualitative studies, in which the goal is to collect usable and pertinent data about a phenomenon of interest from key stakeholders, while permitting interaction, dialogue, and exchange of ideas among participants (Morse, 2000). Eligible participants were pregnant teens between the ages of 13 and 19 years and HPs age 18 or older who currently work with pregnant teens in CS. The study team aimed for 50% of the HPs to be female.

The goal was to answer: (1) What do teens and HPs in CS say about how teens come to experience unplanned pregnancies? (2) Are there heretofore unknown, context-specific factors, that contribute to unplanned teen pregnancies in CS? (3) What specific challenges do teens and HPs say are associated with unplanned pregnancies in CS? and, (4) What specific areas of intervention are needed to address mental health needs of CS teens experiencing unplanned pregnancies?

### Procedures

Prior to the research team’s arrival in Haiti, colleagues at the Organization for the Renovation and Education of the CS Zone (OREZON), a local social service organization with which the research team has had several previous successful collaborations, posted flyers throughout CS. The flyers emphasized the voluntary nature of the study and that a parent's verbal and signed permission were required for teen participants younger than 18, as well as such teens' assent. The flyers contained the names and phone numbers of OREZON staff, who collected volunteers’ names and contact information and assessed eligibility.

Upon arrival in Haiti in December 2018, the primary author, a mid-career native, Haitian female, who is fluent in English and Haitian *Kreyòl*, with three decades of experience as a licensed clinical social worker treating Haitian populations, and over 10 years’ experience as a public health researcher, met with potential participants to confirm eligibility and to obtain informed consent. Teens younger than 18 years provided their assent in the company of a parent who provided signed permission for their daughters to participate in the study. All presenting parents were mothers. Teens’ mothers did not participate in FGs.

Several steps were taken to minimize risk to FG participants. First, prior to each FG, the primary author reviewed elements of the consent document and assessed participants’ informed consent by asking each to summarize in their own words the study’s purpose, benefits, and risks. She then provided information about the study’s purpose, including its benefits and risks and the fact that participants could discontinue participation at any time during the study without risk of losing access to any future intervention. Second, FGs took place at an office in Delmas 33, a location that OREZON colleagues had confirmed as safe, private, and accessible; OREZON colleagues also facilitated round trip transportation for participants. Third, OREZON staff were available to accompany any teens who showed evidence of distress during FGs to local health providers. Fourth, the primary author drew on her professional experience as a Haitian-born licensed clinical social worker to support teens as needed. As an example, when a participant in one of the teens’ FGs became distressed, the primary author moved closer to her, gave her time to cry, and then, when appropriate, interjected, “*Krik*!”, alluding to a familiar and humorous story-telling tradition (Danticat, 1995). At that, all the girls in the FG brightened, leaned forward, and responded “*Krak*!”. A brief traditional story helped participants shift from hurt to laughter and the FG resumed. Other steps taken to minimize risk to the participants was that they could use pseudonyms in lieu of their actual names, and in reporting this study’s findings, participants names have been changed to protect confidentiality. Finally, to insure protection of human subjects in this study, the study protocol and measures were reviewed and approved by the National Bioethics Committee of Haiti’s Ministry of Health and by the Institutional Review Board at the University of South Florida.

We used a Haitian *Kreyὁl* language semi-structured guide with open-ended questions and pertinent prompts to conduct the FGs (Available upon request). FGs lasted 60–90 minutes each and were audio-recorded and then transcribed and analyzed via an inductive coding approach (Corbin & Strauss, 1990). The HPs’ FG was held on the first of three days, followed by the 13–17 year-olds’ and the 18–19-year-old teens’ FGs consecutively. Participants received 25 USD.00 per FG. OREZON colleagues determined that this amount was non-coercive and helpful to teens facing added economic challenges as up-and-coming parents. Participants also received a traditional meal and beverages.

### Analysis

The primary author transcribed the FG audio recordings directly into English and then reviewed each for accuracy by reading the transcripts while listening to the *Kreyὁl* recordings. The unit of analysis was the transcripts of the six FGs, loaded as primary documents into ATLAS.ti, a software for managing, coding, and analyzing qualitative data in a hermeneutic framework (Scientific Software Development, 2013).

Transcripts were first coded using “open coding” in which the primary author and the second author, a mid-career maternal and child health and violence against women expert, highlighted segments of text that reflected experiences of girls and women in CS and assigned *apriori* labels from the pertinent scientific literature to them (Corbin & Strauss, 1990). For example, sections of text were labeled “Poverty and hunger,” “Loss of parents in 2010 earthquake,” “Exposure to neighborhood violence,” Experience of IPV,” and “NPSV.” In view of the literature regarding the mental health of teens experiencing unplanned pregnancies, other segments of texts were given labels such as “Stress,” “Depression,” “Suicidal ideation/gesture,” and “Anxiety.”

As open coding progressed, other segments of text were coded as they related to factors leading to teen pregnancy, associated challenges, and mental health needs. Therefore, at this phase of coding, categories emerged regarding “How teens became pregnant,” “Terminology used to describe teen pregnancy,” “Self-blame,” “Guilt,” “Parental rejection,” “Experience of IPV,” and “NPSV.” As open coding proceeded further, other sections of the transcripts were assigned labels such as “Lack of consistent and accurate sex education,” Lack of knowledge of reproductive rights,” “Banm prèv” translated “Give me proof,” and “Community stigma.” The relationships among the quotes and initial categories were later identified during axial coding, using the Code Family option in ATLAS.ti®.

The Code Family feature permitted the team to group categories and codes such as “Loss of parents in 2010 Haiti earthquake,” Poverty and hunger,” “Lack of consistent and accurate sex education,” and “Banm prèv as a covert form of NPSV” into “Factors contributing to teen pregnancy.” It also permitted grouping of categories such as “depression, guilt, self-blame, anxiety and stigma,” “Mothers’ reactions to teens’ unplanned pregnancies,” “Impact of teens’ mothers’ experiences of anger, shame and depression on teens,” and “Biopsychosocial effects of *banm prèv* pregnancies on teens.” The categories “Parental rejection/abandonment,” “Parental shame, rage and abuse” and “parental depression” were grouped into “Mothers’ reactions to teens’ unplanned pregnancies.”

In selective coding, “Factors contributing to teen pregnancy” were associated with a “Lack of knowledge of reproductive rights,” which, taking into account “Banm prèv as a covert form of NPSV” and “Lack of consent as NPSV,” and their associated codes and quotations, were assigned to “Coercive control _Gendered vulnerability.” Similarly, “Biopsychosocial effects of *banm prèv* pregnancies,” “Devaluation of girls,” and “Community stigma,” “Disparate gender norms and effects of stereotyping,” and their codes and quotations were assigned to “Human rights Opportunity for intervention.”

Saturation in coding occurred when participants’ quotes no longer yielded new information that would enhance understanding of factors leading to teen pregnancy in CS, challenges of such pregnancies and teens’ mental health needs, and when all codes and associated quotations obtained through axial and selective coding were placed into themes that reflected clear answers to our research questions.

## Results

The final sample size was limited by funding restraints, by the nature of the study, and by unanticipated events. For example, a teen recruited for the 13–17-year-old group was forbidden to attend by her boyfriend, even though the mother had signed consent. Also, one of the HPs was ill on the day that the FG was conducted and could not participate. Four of the eight participating HPs were women.

Mean participant age in the 13–17-year-old and 18–19-year-old FGs was 15 and 18.5 years, respectively. Mean participant age of HPs was less than 34 years old. None of the teen participants was enrolled in school at the time of this study either, because their mothers could not afford to continue to pay their tuition as well as assume the additional financial burden of their unplanned pregnancies, or because they were attempting to avoid pregnancy-associated stigma and bullying from school peers. Notably, none of the teens resided with both parents. Only their mothers were responsible for their economic and moral support. All the HPs were employed part-time and none of the teens was employed.

### Factors contributing to teen pregnancy in Cité Soleil

Factors leading to unplanned teen pregnancy in CS included the 2010 Haiti earthquake, poverty/hunger, lack of consistent and accurate sex education, and “*Banm prèv*” translated “Give me proof.”

#### The 2010 Haiti earthquake

All teens and HPs confirmed extant knowledge that the loss of primary caregivers and financial providers in the 2010 earthquake and the lack of parental supervision increased the socioeconomic vulnerability of girls and heightened teens’ risks of being battered, preyed upon, and trafficked. A social worker in the HP FG described the earthquake’s influence on teens’ biopsychosocial susceptibility and on the vulnerability of their unborn children:

The economic situation in CS was already bad. CS got little aid after the earthquake that left orphaned girls in in its wake. These girls started out with little and ended up with nothing. They didn’t get any help to deal with losing parents or homes. When guys beat them, they transpose all that violence on the unborn child.

Marie Dieula, 34

Marie Dieula’s assertions are consistent with previous findings that battered teens are at increased risk of trauma and of battering their own children (Goldenson et al., 2007).

All teens in both FGs confirmed the following testimony from Therèse as familiar and common. Therèse described mistreatment at the hands of a purported female benefactor, who offered her shelter in the aftermath of the earthquake, but who mistreated her and later trafficked her to a man who would beat her, force her into sexual intercourse, and who would continue to beat her even after impregnating her:

I was 9 or 10 when the earthquake happened. My parents died in it. A lady found me and said I could live with her. I spent years as her *restavek* (child slave), rising early to get her kids ready for school and to cook and getting beatings when I refused. When I was 15, she brought me to some man. He forced me to have sex that night and beat me. I thought he’d stop when I got pregnant. He still beats me.

Therèse, 19

Teens and HPs alike also stressed that along with the demand for proof they face from men, poverty, and hunger also contribute to unplanned teen pregnancies in CS, even for girls who do not intentionally use sex as a transaction.

#### Poverty and hunger

Participants in this study underscored a relationship between poverty, hunger, coercive control, and gendered vulnerability. They maintained that teens whose mothers lack financial means fall prey to neighborhood males who provide money for food with expectations of sex in return. Teens reported that hunger is painful and hinders their ability to focus on anything but food.

A social worker in the HP FG described how hunger increases CS teens’ vulnerability to male predators:

As adults, they (men) should help the child anyway, but they insist the child must give something in return. So, sometimes in the search of just a plate of spaghetti to stave off hunger is how young girls become pregnant.

Ronèl, 34

A teen FG participant confirmed (and others concurred) that poverty and hunger interfered with teens’ ability to think and sleep and described the contemplated risks involved:

A poor girl like me, what am I supposed to do when I’m hungry and can’t find food? I can’t think or even sleep when hunger pain strikes my belly! You place your life in anyone’s hands who promises a meal. You worry about danger later, but then it’s too late.

Rosalie, 14

Participants in all three FGs reported that unplanned teen pregnancies also increase the burden and extent of poverty for CS teens. As a participant in the older teens’ FG noted,

Most days, neither you nor your parent have the money to buy even a cup of coffee and a piece of bread, let’s not talk about money for doctors and hospitals. I didn’t expect all these additional costs to a pregnancy. These days, even the cheapest hospitals cost *tèt nèg* (Figuratively, “a man’s head”, i.e., a sacrificial expense). Then, you need money to buy things that you’ll need for yourself and for the baby in addition to money you would ordinarily need to get something to eat and drink. A pregnant person shouldn’t suffer.

Janine, 19

In addition to poverty, hunger, and the loss of primary caregivers, study participants cited the lack of consistent and accurate sex education among factors that led to teens’ unplanned pregnancies.

#### Lack of consistent and accurate sex education

Teens in both FGs reported that discussions about sex were forbidden at home and that sex education was not part of their schools’ curriculum. A social worker in the HP FG noted that teens’ lack of knowledge about how sexual activity and conception are related, combined with their social and emotional immaturity, lead to unplanned pregnancy, guilt, and remorse:

Many teens in CS become pregnant when they enter into sexual activities before their *espri devlope* (development of emotional maturity). They have no knowledge how to prevent pregnancies or exactly how sex relates to pregnancy. They easily become trapped. Later, when they see the consequences of their errors, they feel guilty and say, ‘If only I’d known … ’

Marie Dieula, 34

Marie Dieula’s statement was echoed by other HPs. A participant in the older teens’ FG corroborated that lack of sex education at schools contributed to her pregnancy, but ascribed lack of access to such information to community violence in CS:

Sometimes you have sex before your period comes and you don’t get pregnant, so you think you’re ok; but the school didn’t tell you if it’s before or after you get your period that you can become pregnant. They don’t explain those things. And who’s going to come into CS to teach us? They get paid to come in, but they don’t dare. The female health agents are afraid of getting raped and the males fear being robbed or even killed.

Therèse, 19

The youngest teens may be particularly at risk when they become sexually active as reticence to ask their mothers for information about sex and pregnancy for fear of being castigated, lead to them to rely on uninformed friends:

After my first time having sex, I told a friend. She told me about a pill to prevent getting pregnant. I said I didn’t have money, so she said I can buy less and just take it every three months. I bought it from a street vendor, and was taking it every three months. My mother caught me taking it one morning and yelled at me for doing it. Now, I’m pregnant and my mother criticizes and humiliates me constantly.

Micheline, 16

Despite insufficient and inaccurate messages about sex and reproduction, most teens were familiar with male condoms, a topic that evoked lively discussions in teens’ FGs:

Some guys say that when they use [condoms], the way that they would *‘ὁganize yo’* (handle their business) is messed up (Laughter all around). Yes! The girls also say they don’t enjoy themselves when they use condoms. When they don’t wear them, they feel more at ease. Guys don’t like them. Guys say it feels better without it and they like girls who don’t ask to use them more.

(Excerpts from Younger FG).

None of the teens in the two FGs knew about female condoms, and those who had a history of condom use reported inconsistent use. Other teens shared reasons for nonuse of condoms, including perceptions that condoms cause adverse physical reactions. Rosemite, 15, stated, “The one time … *the one time* (her emphasis) I used one, it made my vagina itch! Since then, I’ve never used those things.”

The reported lack of consistent and accurate sex education and the inconsistent use of condoms in this sample, together with the prevalence of preventable diseases in CS, appear to contribute to the inability of teens (and their parents) to discern symptoms of pregnancy from other conditions. In response to how she became pregnant, a teen from the younger FG said:

I didn’t even know I was pregnant! I didn’t see my period, so I told my mother. She took me to the hospital and they said I probably had typhoid and sent me home, but I didn’t get better. I kept throwing up. When I went back, they did tests for syphilis and AIDS and said I didn’t have those. The third time, they said I was pregnant, that the baby had given me an infection, and that I was anaemic.

Tamara, 14

The lack of accurate and accessible sex education reported by teens seems to render them vulnerable to a contextual risk called *banm prèv*, translated, “Give me proof.”

#### Banm Prèv as covert NPSV

Of the 15 teens in this study, 94% (n = 14) admitted to *Banm prèv* or being forced to give proof of either virginity or non-promiscuity as the origin of their unplanned pregnancies. *Banm prèv* occurs when CS girls are approached and propositioned by males. As the youngest FG participant quietly explained,

When they say *‘Banm prèv’*, if you outright refuse, guys threaten you, or worse, they plan a *kadejak* (translated, “gang rape”) to punish you. They say you must think you look too good or that you’re too educated for them, or they say you’re too proud and they have to humble you. Knowing that, you may be scared to say no and you agree right away to have sex. But, when you do that, they call you “rapid” (quick to give in) and when they’re done with you, they spread the word that you’re rapid, so others come after you. You don’t really have a choice.

Benita, 13

The pressure to provide proof is sometimes craftier, as indicated by a teen from the older FG:

We six all live in one room and sleep on the ground. One night, I woke up and my big brother was touching me, so I yelled. Our little house is so close to all the others and there’re no real windows. A neighbor heard us all yelling and spread a rumor that my brother *pèdi mwen* (lost me, i.e., took my virginity). Everybody started calling me *ras kabrit* (related to goats) because they say only goats have sex with relatives. I knew my brother didn’t have sex with me, but the guy who was spreading the rumor demanded *‘Banm prèv’*, so he would tell people the rumor wasn’t true. I went and I proved it! He’s the one who pèdi mwen! When I told him I got pregnant for him, he spit on me and said, ‘Away, *ras kabrit*! Your brother’s the father!

Chedeline, 19

HPs in this study noted that the *banm prèv* phenomenon exists and persists, in part, because of CS gender norms that empower males and render girls and women vulnerable to aggression and maltreatment as well as to coercion. A physician noted and others agreed,

In CS, girls are always victims of one thing or another! Boys have more liberty than girls and boys don’t get consequences. Parents say, *‘Se ti kòk mwen genyen, zafè ti poul nan poulaye pa gade’m*’ (I have a little rooster, what do I care about little chicks in the coop?). They call the pregnant girls names, but congratulate the boy with, *‘Ey*! You’re a man now, eh?’

Elifort, 37

### Biopsychosocial effects of banm prèv pregnancies on teens

HPs noted that girls who fall victim to the *‘Banm prèv’* phenomenon experience sadness and guilt, and at times demonstrate suicidal gestures because although they are deceived or coerced into giving proof of whatever it is a neighborhood male might demand proof of, teens are under the illusion that they consented. A social worker indicated,

These girls (who yield to banm prèv) sit down and cry all day. Since they neither fought back nor screamed out nor got gang-raped, they feel they must be guilty for consenting. The community also holds that view; so, they don’t know what to do and they don’t see a way out. They commonly resort to drinking bleach or rat poison so they can die, because they are *a bout* (Translated, “reached their limits”).

Ronèl, 34

A physician from the HP FG reported insomnia, anxiety, and intrusive symptoms such as nightmares among pregnant teens in her care, who sometimes have been victims of gang rapes as well as *banm prèv*, remarking a sense of hopelessness for their recovery:

These girls have ugly dreams that trouble them. One girl doesn’t know if she’s pregnant from a gang rape or a *banm prèv*. She doesn’t sleep for fear of nightmares. Their lives never turn out well; they live from crisis to crisis.

Ediane, 38

A psychologist from the HP group added that girls who become pregnant from *banm prèv* have a greater risk of depression when, in addition to the challenges noted above, they face stigma and seemingly unbearable burdens of guilt, shame, and self-blame:

These girls who get trapped in the *banm prèv* thing, it’s confusing for them because they think they wanted the sex since they agreed to give proof. They blame themselves on top of the blame they get from society, but they’re never quite sure why they’re blaming themselves.

Marie Dieula, 34

Moreover, because teens are not at liberty to discuss the way they were deceived into sexual activity with parents, once pregnant, they face trauma associated with not only the *banm prèv* but also with parental rejection, abandonment, and abuse.

#### Mothers’ reactions to their teens’ unplanned pregnancies

A participant from the older teens’ FG lamented that her mother’s reaction to her unplanned pregnancy resulted in her being homeless, a situation that causes her such fear, shame, and anxiety that she wishes she had been a victim of a gang rape instead. Her statement reveals that even though *banm prèv* is, in fact, coercive, she erroneously believes that she actually consented:

My mother put me out when she saw I was pregnant. The load of shame I carry that is this pregnancy is heavy as a sack of salt because I agreed to giving proof. If only I had been a victim of *kadejak* (gang rape) instead! At least my mother would’ve had compassion. But, I go from house to house looking for a place to sleep, like a dog. When people see your own mother abandon you, they mistreat you even more.

Gaelle, 18

A participant in the younger teens’ FG reflected that she is not only alienated but abused by her mother because her pregnancy did not result from a gang rape:

When my mother found out I was pregnant, she insisted I tell her the guys’ names who did the *kadejak* (gang rape) on me. I said I wasn’t raped. As long as I live, I’ll never forget the look on her face! I wish she’d killed me instead! She didn’t put me out, but when I ask her for food, she throws the food on the floor at me and yells, ‘Did I send you to get pregnant?’ She shoves and hits me too.

Rosemite, 15

HPs explained that the mothers of pregnant teens also bear a burden of social stigma and shame when their daughters are pregnant, resulting in parental depression.

#### Impact of teens’ mothers’ experiences of anger, stigma, and depression

HPs described that when teens in CS face an unplanned pregnancy, mothers react with shock and anger regarding the pregnancy itself, and, with shame in reaction to community stigma against them as mothers of pregnant teens. Ultimately, these emotions have dire consequences for the health and wellbeing of the pregnant teen and her unborn child. A social worker noted,

[Mothers] are so ashamed when their young girls get pregnant! Everyone criticizes them as bad mothers. Such things worsen the mothers’ misery. They become dazed with rage. They forget that the girl or even the unplanned baby might get them out of poverty someday. A girl’s mother beat her so much the teen had eclampsia and died giving birth. The baby also died three days later.

Ronèl, 34

A nurse midwife in the HP FG confirmed that teens’ mothers not only become angry when their daughters experience an unplanned pregnancy but become depressed. She added that mothers’ reactions adversely impact the physical health of the teens and their fetus as well as the mother–daughter relationship:

Sometimes, the teens’ mothers become so depressed, they want to kill themselves as well as their daughter! They find their girl is pregnant, they think too much of nothing else all day long, and it becomes a big problem! It’s very difficult for them to show [teens] affection or support them because they are suffering themselves.

Guerline, 29

## Discussion

A key contribution of this study is in its identification of a coercive but covert form of gender-based NPSV that greatly contributes to unplanned teen pregnancy in CS. The potential impact of this study was supported by psychologists at Partners in Health in March 2019 meetings in which they revealed that *banm prèv* is also common in rural Haiti (Personal communication, E. Eustache, and team of psychologists, March 2019).

The *banm prèv* phenomenon was a common and direct cause of pregnancies in our sample. It appears to be an accepted, contextualized norm that renders teens vulnerable to covert, coercive control of their bodies, with no apparent right to dissent. It takes away teens’ rights to agree to sexual interaction in a timeframe that they find acceptable. It also reflects the violation of teens’ rights to bodily integrity, to personal autonomy, to deciding when to be sexually active, and with whom, and to choosing when and how to get pregnant (The Lancet Commissions, 2018).

The *banm prèv* phenomenon underscores that violent maltreatment of girls and women in CS is not limited to domestic violence (DV), IPV, or NPSV, but encompasses a constant threat of gender-based aggression and entrapment that takes advantage of teens’ inability to discern consensual from coerced sex. Furthermore, the confluence of *banm prèv*, poverty, hunger, IPV, and parental abuse (DV) when pregnant is concerning as female victims of IPV, DV, and NPSV are at a greater risk for depression, anxiety, insomnia, and stress-related disorders compared to male victims.

There are preventive measures for *banm prèv*. For example, the WHO has outlined specific “recommendations on adolescent sexual and reproductive health and rights [including] issues that may be important for the human rights, health and well-being of adolescents … in a user-friendly” format (WHO, 2018b, p. 9). Collaboration with the Haitian Ministry of Health and community leaders in CS could enable implementation of its guidelines and could mitigate the occurrence of NPSV and *banm prèv*, especially if girls as well as boys are targeted for intervention. Moreover, community training aimed at stigma reduction could aid in reducing self-blame, anxiety, and trauma among pregnant teens. Providing sensitivity training to teachers and school children would also go a long way toward enabling teens to remain in school while pregnant and to reach their full personal socioeconomic, and emotional potential (UNFPA, 2018).

The existence and passive acceptance of the b*anm prèv* phenomenon illustrates that assigning Haitian girls and women the gender-based role of *poto mitan* (load bearing center post of a structure) essentially sets them up to experience personal, psychological, and social distress that, without intervention, can lead to intergenerational trauma. Findings support Lamour’s assertion that the *poto mitan* label for Haitian women is an illusion and perhaps even a deception designed to perpetuate their vulnerability (Lamour, 2019). It would be important to have open town hall meetings in which teens and their parents verbalize to local policymakers and international donors the burdens associated with their presumed roles as poto mitan of the Haitian family. In that context, teens and their parents could express what they need to truly fulfil that role, i.e., socioeconomic, and psychological support that would enable them to combat personal poverty and hunger and reduce their susceptibility to predators or coercion.

## Conclusion

Our findings indicate that despite the tremendous efforts of NGOs such as SOFA (Solidarity of Haitian Women), USAID, UNFPA, and CS community leaders, *banm prèv* is a new dimension of human rights violation that exists for teens in CS; it contributes to unplanned pregnancies and increases risks of depression, suicidality, and trauma. Disruption of the *banm prèv* phenomenon must begin at the community level, since even with comprehensive sexuality education and knowledge of their rights, girls in CS would not be able to safely refuse sex. Since “developing and strengthening [teens’] capacity to reduce and manage risk is not a secondary … concern but an essential first step in the hard work ahead of building more … resilient communities” (Enarson, 2002, p. 3), we felt it was a moral/ethical imperative to begin disrupting the *banm prèv* norm in CS as a first step in reducing unplanned pregnancies and in informing the community of girls’ and women’s rights to safely refuse sex. Therefore, in partnership with OREZON (mentioned above), we selected 300 Cité Soleil male and female teens aged 13–19 (including teens who had not participated in our study) as well as adults, to participate in a *banm prèv* awareness and prevention campaign. The campaign included live skits using the *krik krak* storytelling tradition. Topics of the skits included girls’ and women’s right to safely refuse sex, why a young man should not demand proof of virginity/humility or commit NPSV, the impact of *banm prèv* and NPSV on girls and on the community, and the damaging effects of community stigma toward teens experiencing an unplanned pregnancy on them and on their mothers. The event was intentionally scheduled for November 25, 2019, the International day for Prevention of Violence against Women. Attendees provided very positive feedback. We are seeking funding to maintain a sustained effort to combat *banm prèv* in CS and in adjacent shantytown.

To begin increasing teens’ agency, we trained 60 girls to make shampoo, hair conditioner and laundry detergent and to bake bread and cupcakes, as there is a market for such items in Cité Soleil. This would enable them to step toward economic self-sufficiency as a path to reducing their socioeconomic vulnerability.

## Limitations

This was a small, exploratory study of key stakeholders. A larger sample may have yielded expanded or different information, particularly regarding the impact of education on teens’ vulnerability to *banm prèv*. Findings are not generalizable to other pregnant teens in CS, in Haiti, or in other LMICs. Nevertheless, the study has several strengths. The primary author is a co-ethnic researcher, who is fluent in Haitian *Kreyòl* and who has had successful collaborations with OREZON, a trusted social service agency in CS. Together, they are familiar with the social, political, economic, and cultural context of CS. Third, the research team obtained perspectives of key stakeholders (teens and HPs) whose personal strength and intelligence are reflected in the carefully detailed and pensive perspectives that they offered.

### Implications for practice

The Haitian culture has many strengths and protective factors, including religion, faith, spirituality, and a long-standing tenet of children as riches of the poor (Jeanty & Brown, 1976). However, as described in this study, these factors might be difficult to call upon due to biopsychosocial stressors. An important step would using traditional strengths to intentionally dispel patriarchal norms which heighten girls' and women's vulnerability and promote messages of gender equity, human rights, rape empathy, and charity. Follow-up events to the *banm prèv* prevention campaign that we conducted will include such efforts. Moreover, UNFPA and the Haitian Ministry of Health could add *banm prèv* as a contextual, calculated, and coercive risk for girls and women when preparing comprehensive sexuality curricula and add civic and moral education for young boys and men that support and empower girls and women in a context of respect and collaboration. UNFPA could collaborate with SOFA and other organizations that advocate for girls’ and women’s human rights to expand services and include victims of *banm prèv* as well as victims of overt NPSV. Such efforts would reduce the obscurity of what constitutes consent, reduce the likelihood of being a perpetrator or victim of *banm prèv* and reduce the burden of unplanned teen pregnancy in Haiti.

### Human rights: Opportunity for intervention

Addressing human rights for disadvantaged populations, Patel & Farmer submit that “The benefits of high-quality health care must be made available and accessible to all people, irrespective of their social station or where they live” (Patel & Farmer, 2020, p. 108). To our knowledge, no current interventions in CS address the biopsychosocial health needs of Haitian teens impacted by multiple stressors and traumas in addition to an unplanned pregnancy. There is scientific evidence that mental health support and skills training can provide young mothers social support, increase their parenting skills, and decrease their symptoms of depression and trauma (Muzik et al., 2016). Research supports the use and efficacy of videos that are culturally relevant and that reflect similar actors or presenters to deliver interventions for Haitian youth (Malow et al., 2009). Colleagues at OREZON had previously agreed that conceptually, using *Krik-Krak* as a framework for developing a trauma-informed intervention for pregnant teens was likely to be engaging.

The *krik-krak* story-telling format was powerful in deescalating a teen participant, in engaging her and her peers, and in helping them make a cognitive shift from hurt and sadness. Using that format, scripts can be developed based on participants’ expressed contextual challenges. Information that promotes body autonomy and human rights, and helps teens discern between consent and coercion (however covert) would be an important component of sex education and reproductive rights for teens. In addition, teens’ challenges could be integrated into videos that teach them how to combat stress, manage DV or IPV during pregnancy, and cope with shame, guilt, and self-blame. Additional videos could be developed for purposes of community education, in which NPSV is distinguished from *banm prèv* while highlighting similarities between the two; it would intentionally target young boys and girls and would include messages that emphasize the difference between consent and succumbing to coercion. Other audiences that could be targeted in a *krik-krak* video intervention include parents, teachers, local HPs, and community members in efforts, with messages aimed at reducing stigma, increasing social support for teens, and preventing parental violence, humiliation, and abandonment of pregnant teens. If successful, these video interventions would be an important step toward mitigating household, neighborhood and personal stress and depressive symptoms of teens.

Finally, future studies should include teens experiencing unplanned pregnancies together with their mothers with the aim of reducing stress for both, strengthening bonds of affirmation and solidarity between them and working together to avoid transmitting the illusion and burden of girls and women as the *poto mitan* or load-bearing post of the Haitian household. This would empower mothers and daughters and reduce maternal shame, guilt, and trauma across generations of Haitian women.
